# Observational studies - should we simply ignore them in assessing transfusion outcomes?

**DOI:** 10.1186/s12871-016-0264-4

**Published:** 2016-10-14

**Authors:** Kevin Trentino, Shannon Farmer, Irwin Gross, Aryeh Shander, James Isbister

**Affiliations:** 1 Performance Unit, South Metropolitan Health Service, Perth, Western Australia Australia; 2 School of Surgery, Faculty of Medicine Dentistry and Health Sciences, The University of Western Australia, Perth, Western Australia Australia; 3 Centre for Population Health Research, Faculty of Health Sciences, Curtin University, Perth, Western Australia Australia; 4 Accumen LLC, San Diego, CA USA; 5 Department of Anesthesiology and Critical Care, Englewood Hospital and Medical Center, Englewood, NJ USA; 6 Sydney Medical School, Sydney, NSW Australia

**Keywords:** Randomized controlled trials, Observational studies, Bias, Confounding, Blood transfusion, Causation

## Abstract

**Background:**

As defined by evidence-based medicine randomized controlled trials rank higher than observational studies in the hierarchy of clinical research. Accordingly, when assessing the effects of treatments on patient outcomes, there is a tendency to focus on the study method rather than also appraising the key elements of study design. A long-standing debate regarding findings of randomized controlled trials compared with those of observational studies, their strengths and limitations and questions regarding causal inference, has recently come into focus in relation to research assessing patient outcomes in transfusion medicine.

**Discussion:**

Observational studies are seen to have limitations that are largely avoided with randomized controlled trials, leading to the view that observational studies should not generally be used to inform practice. For example, observational studies examining patient outcomes associated with blood transfusion often present higher estimates of adverse outcomes than randomized controlled trials. Some have explained this difference as being a result of observational studies not properly adjusting for differences between patients transfused and those not transfused. However, one factor often overlooked, likely contributing to these variances between study methods is different exposure criteria. Another common to both study methods is exposure dose, specifically, measuring units transfused during only a part of the patient’s hospital stay.

**Summary:**

When comparing the results of observational studies with randomized controlled trials assessing transfusion outcomes it is important that one consider not only the *study method*, but also the key elements of *study design*. Any study, regardless of its method, should focus on accurate measurement of the exposure and outcome variables of interest. Failure to do so may subject the study, regardless of its type, to bias and the need to interpret the results with caution.

## Background

### What is the current problem?

The results of randomized controlled trials (RCTs) are not always consistent with the results of observational studies, often leading to much debate [1–3]. RCTs rank higher in the hierarchy of clinical research than observational studies and this could lead some to assume that observational studies should not be used to inform practice [4]. Some have suggested that observational studies overestimate treatment effects and provide misleading conclusions [1, 5–7].

### What is the prejudice against observational studies?

It is generally believed that observational studies cannot establish causation. They are considered susceptible to unidentified confounders and prone to overestimate treatment benefit and harm. Different results between RCTs and observational studies are cited as examples.

### Are the prejudices justified?

In a two-part series of articles in The New England Journal of Medicine the authors compared results from RCTs with observational studies. Interestingly, neither article found evidence to support the claim that the results of observational studies are inferior to, or likely to vary widely from, the results of RCTs [4, 8].

Dismissing observational studies based on study type (or study method) alone is short-sighted. The view that observational studies play no role in establishing causality is invalid, especially when conducting an RCT is impossible (e.g. smoking vs no smoking), unethical (e.g. comparing transfusion with no transfusion in critical haemorrhage), logistically impossible (eg a very large trial investigating all-comers regardless of age and multiple co-morbidities and multiple intervention outcomes), or equipoise cannot be clearly established. This is the case when it comes to assessing patient outcomes associated with blood transfusion.

This article presents often overlooked factors likely to contribute to the difference in results of observational studies compared with RCTs evaluating the outcomes of blood transfusion.

## Discussion

### How do observational studies compare with RCTs in evaluating the outcomes of blood transfusion?

In 2008 a systematic review of the literature was conducted to determine the relationship between red blood cell (RBC) transfusion and patient outcomes in critically ill patients. The pooled results from the observational studies found that RBC transfusion was independently associated with higher odds of mortality and higher odds of developing an infection [9].

The pooled results from these observational studies were different from the pooled results from RCTs [10, 11]. For example, the meta-analysis of observational studies found a 1.7 times higher odds of mortality in transfused patients (95 % CI = 1.4–2.2) whereas a Cochrane review of RCTs found that liberal transfusion was associated with a 1.3 times higher risk of mortality (95 % CI = 1.1–1.6) [12]. The pooled results from the observational studies also found a 1.8 times higher risk of infection in transfused patients (95 % CI = 1.5–2.2), higher than the risk of infection with liberal transfusion reported in the Rhode et al. review of RCTs (RR 1.2; 95 % CI = 1.1–1.4) [13].

### What is the reason for the apparent difference?

A reason commonly presented to explain this difference is that observational studies are prone to bias because transfused patients are sicker and therefore more likely to have poorer outcomes [14, 15].

Many observational studies highlight that, on average, patients transfused are older and have more comorbidities than patients not transfused. Few, if any, would doubt that unless severity of illness is adjusted for, results will be confounded. However, there are two main reasons why the observed differences between the results of observational studies and of RCTs are not completely explained by patients being sicker.

First, the observational studies included in the review consistently adjusted for confounders [9]. For example, results of 12 different studies were pooled in the mortality analysis. All 12 of these pooled studies adjusted for confounders (Table 1). These studies adjusted for a total of 167 confounders (median = 9) with a range of 3–30 confounders. When analysing the confounders included in the 12 pooled studies, 11 included age, 10 included gender and 6 included race. All studies attempted to adjust for the ‘sicker’ patient. For example, all 4 of the mortality studies in trauma adjusted for the Injury Severity Score and 3 of the 4 included the Glasgow Coma Scale. To account for differences in patients presenting for isolated coronary artery bypass graft (CABG) surgery, researchers from the Cleveland Clinic included 29 confounders “known to be associated with adverse outcome after CABG” in their analysis.

**Table 1:** Adjustments included in the 12 pooled observational studies into RBC transfusions relationship with mortality

	Studies analyzed listed by first author												
Confounder	Carson	Corwin	Croce	Dunne	Gong	Janson	Koch	Malone	Rao	Silver-board	Vincent	Yang	Total
Cardio-vascular comorbidities	6		1		1		9		13			13	43
Demographic/Socio-economic factors	5		2	3	3	2	3	3	4	2	1	5	33
Severity of disease scores	3		3	2	1	1	1	3	1	1	2		18
Other admission adjustments	6				3	1			1	1			12
Perioperative adjustments	3					3	5						11
Tests and procedure results	1		1		3		2	2	1				10
Hemoglobin/Hematocrit	2	2					1	1	1		1		8
Diabetes					1		2		1			1	5
Alcohol/Tobacco use	1				2		2						5
Medication	1				1				3				5
Renal comorbidities					1		1		1			1	4
Smoking status									1			1	2
Blood loss	1					1							2
Family history of disease									1			1	2
Other blood products							2						2
Respiratory comorbidities							1						1
Liver comorbidities					1								1
Pre-operative transfusion	1												1
Age of blood transfused		1											1
Septic shock					1								1
Total	30	3	7	5	18	8	29	9	28	4	4	22	167

Second, it isn’t always the sicker patient who is transfused. Large, unexplained variability in transfusion practice exists and has persisted for some time [16]. The decision to transfuse is often based simply on a hemoglobin value predetermined by the physician (independent of whether the patient is male or female, despite having a different normal hemoglobin value), without regard to the clinical or morbid condition of the patient [17]. One study found that chronic kidney disease alone, in the absence of anemia, was not an independent predictor of transfusion in patients undergoing major joint arthrosplasty. However, a low hemoglobin level was independently associated with increased transfusion utilization [18]. There are also differences in transfusion rates by gender [19]. Additionally, a number of studies have shown that transfusion is associated with worse outcomes in less sick patients compared with those who are more sick [20, 21]. For example, Ferraris and colleagues, assessing transfusions and adverse surgical outcomes, found that patients at low risk for surgical morbidity or mortality had between an 8- and 10-fold excess risk of adverse outcomes when they received a blood transfusion compared to high risk patients [22]. These factors, and others discussed elsewhere [23], suggest that the “sicker patient” hypothesis alone is not sufficient to explain the differences between study types and dismiss the findings of observational studies. Most importantly, no one has identified the “missing” co-morbidity of the “sicker patient” to explain the differences in outcome between transfused and non-transfused patients.

### How do the differences in exposure criteria affect the analysis of results?

Another factor that may contribute to differences between pooled results of the observational studies and the RCTs is that different exposure criteria were studied. Hatala and colleagues described 4 key elements of study *design*, namely (1) patients, (2) interventions (exposure), (3) outcomes and (4) study methods [24]. The exposure studied in the review of observational studies differed from the exposure studied in the review of RCTs (Table 2). The meta-analysis of observational studies sought to estimate the association between ***transfusion versus no transfusion*** and outcomes. The meta-analysis of RCTs looked at a ***liberal versus restrictive*** transfusion threshold and outcomes. This is a critical distinction [4].

**Table 2:** The four key elements of study design applied to three systematic reviews of transfusion literature

Study	1. Patients	2. Intervention/Exposure	3. Outcomes	4. Study methods
Efficacy of red blood cell transfusion in the critically ill: A systematic review of the literature	Cardiac Patients	Comparison between patients transfused red blood cells to patients not transfused red blood cells	Mortality	Observational Studies
	Critical Care Patients		Infection	
	Orthopaedics		Multiorgan Dysfunction	
	Trauma		Acute Respiratory Distress	
	General Surgery			
	Neurosurgery			
Transfusion thresholds and other strategies for guiding Transfusion thresholds and other strategies for guiding red blood cell transfusion (Cochrane Review)	Cardiac Patients	Comparison of patients assigned to a liberal transfusion strategy to patients assigned to a restrictive transfusion strategy	Mortality	Randomized Controlled Trials
	Critical Care Patients		Infection	
	Orthopaedics		Length of Stay	
	GI Bleeding		Function and Fatigue	
	Trauma		Cardiac events	
	Vascular		Myocardial infarction	
	Haematology		Pulmonary oedema	
			Stroke	
			Pneumonia	
			Thromboembolism	
			Rebleeding	
			Renal failure	
			Mental confusion	
HealthCare–Associated Infection After Red Blood Cell Transfusion: A Systematic Review and Meta-analysis	Cardiac Patients	Comparison of patients assigned to a liberal transfusion strategy to patients assigned to a restrictive transfusion strategy	Infection	Randomized Controlled Trials
	Critical Care Patients			
	Orthopaedics			
	GI Bleeding			
	Post Partum			
	Sickle Cell			

Adapted from Hatala R, et al. Tips for learners of evidence-based medicine: 4. Assessing heterogeneity of primary studies in systematic reviews and whether to combine their results. CMAJ : Canadian Medical Association journal 2005;172(5):661–5

A 2015 systematic review and meta-analysis assessed the outcomes of RCTs compared with observational studies in cardiac surgery [25]. The forest plots for RCTs and observational studies are both labelled “liberal transfusion” vs “restrictive transfusion”. This could lead the casual reader to conclude that both study methods used the same exposure criteria. However, that is not the case. The discussion states that the observational studies compared transfusion with no transfusion. This is not liberal vs restrictive. The accompanying editorial makes the point that “These two assessments are not related in any meaningful way [26].”

In simple terms, observational studies compare outcomes in a group with a 100 % transfusion rate *versus* those with 0 % transfusion. In restrictive *versus* liberal transfusion RCTs, both groups contain a significant number of patients exposed to transfusion. For example, 17 of the 19 trials included in the Cochrane Review referred to earlier provided data on the proportion of patients transfused in both exposure groups. The patients assigned to a liberal transfusion strategy had a pooled transfusion rate of 84 %; patients in the restrictive transfusion group had a transfusion rate of 46 %.

Given this key difference in study design one would expect differences in results. One might expect that the meta-analysis of observational studies, where the difference in transfusion rates between the two groups was 100 %, would produce a higher estimate than any study comparing liberal to restrictive transfusion, where the difference in transfusion rates is always less.

### Are randomized controlled trials free from bias by design?

An assumption often made is that any inherent confounding found in observational studies can be avoided in RCTs [14, 27]. It is true that confounding can often be limited through an RCT, but does this mean that all RCTs are automatically free from bias?

In the pooled results from eleven trials comparing 30-day mortality, two large studies - Transfusion Requirements in Critical Care (TRICC) and Liberal or Restrictive Transfusion in High Risk Patients after Hip Surgery (FOCUS) - contributed 75 % of the weight in the mortality analysis [12].

These two trials reported consistent results - no statistically significant difference in mortality between the two groups studied. However, these trials both recorded a large number of RBC units transfused **prior** to randomization. In the TRICC trial 1045 units were transfused before the 418 patients were assigned to a restrictive strategy, and 966 units to the 420 patients eventually assigned to the liberal. In the FOCUS trial 531 units were transfused before the 1009 patients were assigned to a restrictive strategy, with 452 units to the 1007 patients eventually assigned to the liberal group. These units are not included in the analysis of the relationship of transfusion to outcomes and could potentially introduce an inaccurate estimate of the exposure’s effect on the risk of outcome.

Notwithstanding, these trials can answer important questions. For intensive care physicians the TRICC trial answers the question: Regardless of transfusions that occur prior to the ICU, what might the impact be of a restrictive transfusion threshold while in the ICU on overall outcomes?

However, units transfused prior to randomization, and any units transfused after discharge from ICU, limit the generalization of outcome results. One cannot extend the results and conclude that there is no difference in patient outcome using a restrictive *versus* liberal transfusion threshold in the patient’s entire episode of care. Nor can one use these RCTs to compare the outcomes of transfusion *versus* no transfusion.

One recent trial comparing liberal *versus* restrictive transfusion thresholds did report a statistically significant higher risk of death in the liberal transfusion group [28]. This trial, studying patients with acute gastro-intestinal bleeding, found an 82 % higher risk of death in the liberal group within 45 days. This trial was different from both the TRICC and FOCUS trials in at least one key way: patients were randomized *immediately* after admission and anyone transfused within the previous 90 days was excluded.

Observational studies can also introduce bias from inaccurate measurement of exposure variables. A study of patients undergoing cardiac surgery compared outcomes of those transfused with those not transfused [29]. Transfusion was defined as blood administered within 24 h of admission to ICU. Data suggest that 27 % of patients undergoing cardiac surgery are transfused only in the operating room [30] and it is possible that other patients were transfused only outside the defined 24 h window. So this study design likely misclassified a significant number of patients who received transfusion as “not transfused”.

Any study, whether observational or RCT, that measures only the units transfused during a subpart of the patient’s hospital stay, should be analyzed with caution. This includes the many studies sourced from databases that do not capture all transfusions a patient receives during their hospitalization (e.g. studies using the ACS – NSQIP database). Attempts to generalize the results to transfusion’s association with overall outcomes will likely underestimate the relationship.

RCTs have a number of limitations including tightly defined patient populations, modest sample sizes and short duration outcome measures [31]. Regarding the latter, the Iowa trial found higher adverse neurological events with restrictive transfusion thresholds in preterm infants compared with liberal thresholds [32]. However, a follow-up study of these infants at school age appeared to contradict the early findings, demonstrating long-term adverse effects on neurocognitive and academic function associated with preterm liberal transfusion thresholds [33].

Large prospective observational (phase 4 real-world) studies play a major role not only in generating hypotheses but unequivocally adding new knowledge to the scientific literature. They assess large numbers of diverse real-world patients with short-, mid- and long-term follow-up. RCTs may be sufficient to establish efficacy of a new intervention, but are often not large enough to clearly identify harm [34]. Quality observational studies use a variety of sophisticated statistical analyses, such as multi-variable regression modelling and propensity score matching, in an effort to minimize the potential for confounding. These studies, which control for differences in patient groups, should not simply be dismissed as biased but analyzed on their scientific merit. Sir Austin Bradford Hill, pioneer of the RCT, along with Sir Richard Doll, established the causal link between tobacco use and lung cancer without an RCT [35] and subsequently proposed 9 criteria for establishing causation from association. Isbister and colleagues applied this to observational literature on transfusion outcomes and found that all criteria were met in relation to adverse outcomes associated with transfusion [36].

This issue is timely. “The naysayers continue to believe that only if they could find that one lost confounder that the multitude of agreeing papers would be discredited, somehow making small transfusions an improvement to outcome [37].” These recent comments by Spiess highlight a recurring theme in attitudes dismissive of the findings of observational studies.

## Conclusions

Observational studies examining the outcomes associated with transfusion often present higher estimates of adverse events than RCTs. Some have argued this difference is because of the assumption that sicker patients are transfused and observational studies cannot properly adjust for this difference.

This explanation ignores other factors. It is not always the sicker patient who is transfused. The difference between exposure criteria in observational studies and RCTs limit comparison. Both observational studies and RCTs measuring units transfused only during a subpart of the patient’s hospital stay introduce bias.

Both RCTs and observational studies have strengths and limitations and can be complementary in assessing treatment effects. However, when comparing the results of studies it is important the dialogue focuses not only on the *study method*, but also on the key elements of *study design*. Any study, whether RCT or observational, should focus on accurate measurement of the exposure and outcome variables of interest. A failure to do so potentially exposes the study, regardless of its type, to bias and the need to interpret the results with caution.

## Abbreviations

CABG: Coronary artery bypass graft
FOCUS: Liberal or restrictive transfusion in high risk patients after hip surgery study
ICU: Intensive care unit
RBC: Red blood cell
RCTs: Randomized controlled trials
TRICC: Transfusion Requirements in Critical Care Study
